# A Rare Co-occurrence of Williams Syndrome and 𝘛𝘕𝘒2 Gene-Related Epilepsy

**DOI:** 10.7759/cureus.70777

**Published:** 2024-10-03

**Authors:** Sumathi Angel, Badiginchala Naga Jyothi, Chinthalapalli Prakash Ravikumar, Parag M Tamhankar

**Affiliations:** 1 Pediatric Neurology, Aster CMI Hospital, Bengaluru, IND

**Keywords:** antiepileptic drug, dysmorphic facies, epilepsy in children, exome sequencing, williams-beuren syndrome

## Abstract

Williams syndrome is a multisystem disorder characterized by developmental delay, characteristic facial features, growth abnormalities, and cardiovascular abnormalities. The disorder is an autosomal dominant genetic syndrome that occurs due to microdeletion at chromosomal locus 7q11.23. Seizures occur uncommonly in association with Williams syndrome. Mutations in the *TNK2* gene have been found in rare cases of autosomal recessive infantile-onset epilepsy. We describe a rare co-occurrence of Williams syndrome and *TNK2* gene-related epilepsy in a child born of consanguineous parents. This case report emphasizes the role of genetic testing in the diagnosis of rare diseases. This is the fourth case report of epilepsy with biallelic mutations in the *TNK2* gene, to the best of the authors’ knowledge.

## Introduction

Williams syndrome is a multisystem disorder characterized by developmental delay, characteristic facial features resembling elfin facies, growth abnormalities, and cardiovascular abnormalities. The disorder is sporadic in most families and shows autosomal dominant inheritance. The causative chromosomal mutation is a heterozygous microdeletion of around 1.4 to 1.8 megabases at chromosomal locus 7q11.23. The diagnosis can be made on fluorescent in situ hybridization, chromosomal microarray, multiplex ligase-dependent probe amplification, quantitative polymerase chain reaction, or copy number algorithms of whole exome sequencing (next-generation sequencing technology) [1,2]. Seizures occur uncommonly in association with Williams syndrome and atypical larger deletions are considered to be causative [3,4]. *TNK2 *gene-related autosomal recessive epilepsy is a rare entity that has been reported in only two publications previously [5,6]. To date, three families with homozygous/compound heterozygous variants in the *TNK2 *gene and epilepsy have been described in medical literature [5,6]. The *TNK2 *gene-related neurodevelopmental disorder is characterized by infantile-onset epilepsy, intellectual disability, speech, and cognitive delay. The *TNK2 *gene encodes a cytosolic, nonreceptor tyrosine kinase that shows high expression in the brain. The gene is upregulated during brain development and is expressed in proliferative areas and migratory pathways in the developing brain. We report an infant with clinical features of Williams syndrome and *TNK2 *gene-related epilepsy who was diagnosed on exome sequencing which simultaneously diagnosed both the microdeletion and *TNK2 *gene variant in the patient.

## Case presentation

**Figure 1:**
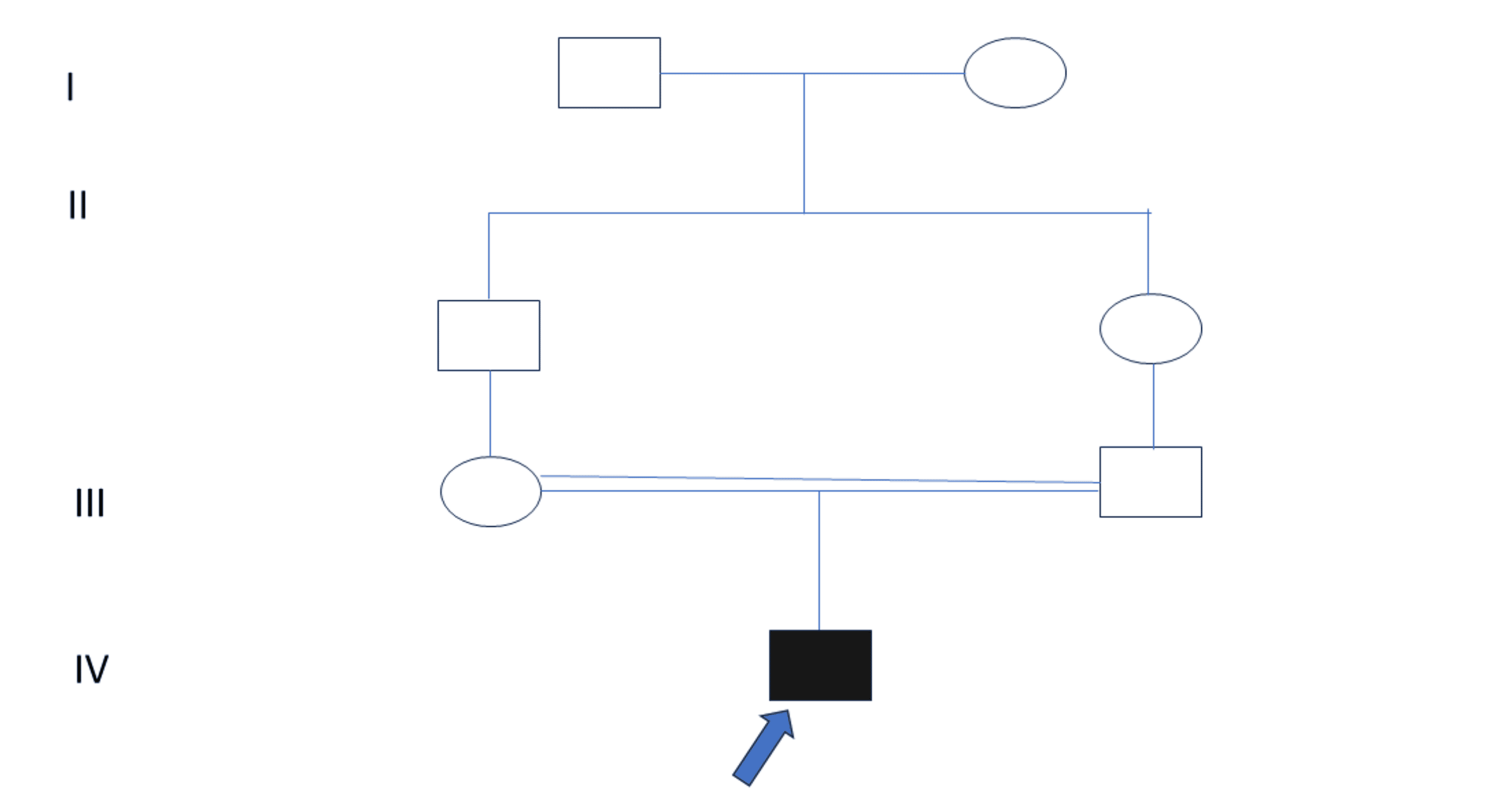
Four-generation pedigree of the family The parents were married in a third-degree consanguineous union, the index child was first born (shown as a darkly shaded box with a blue arrow), boxes represent males, circles represent females, the horizontal connecting line indicates marriage, double horizontal connecting lines indicate consanguineous marriage, the vertical connecting line indicates parent-child relation.

The male patient presented at 10 months of age with developmental delay till 6 months of age followed by regression following the onset of epilepsy. He was born of third-degree consanguineous marriage at term, and his birth weight was 2.5 kg with no major antenatal complications (pedigree shown in Figure 1).

**Figure 2:**
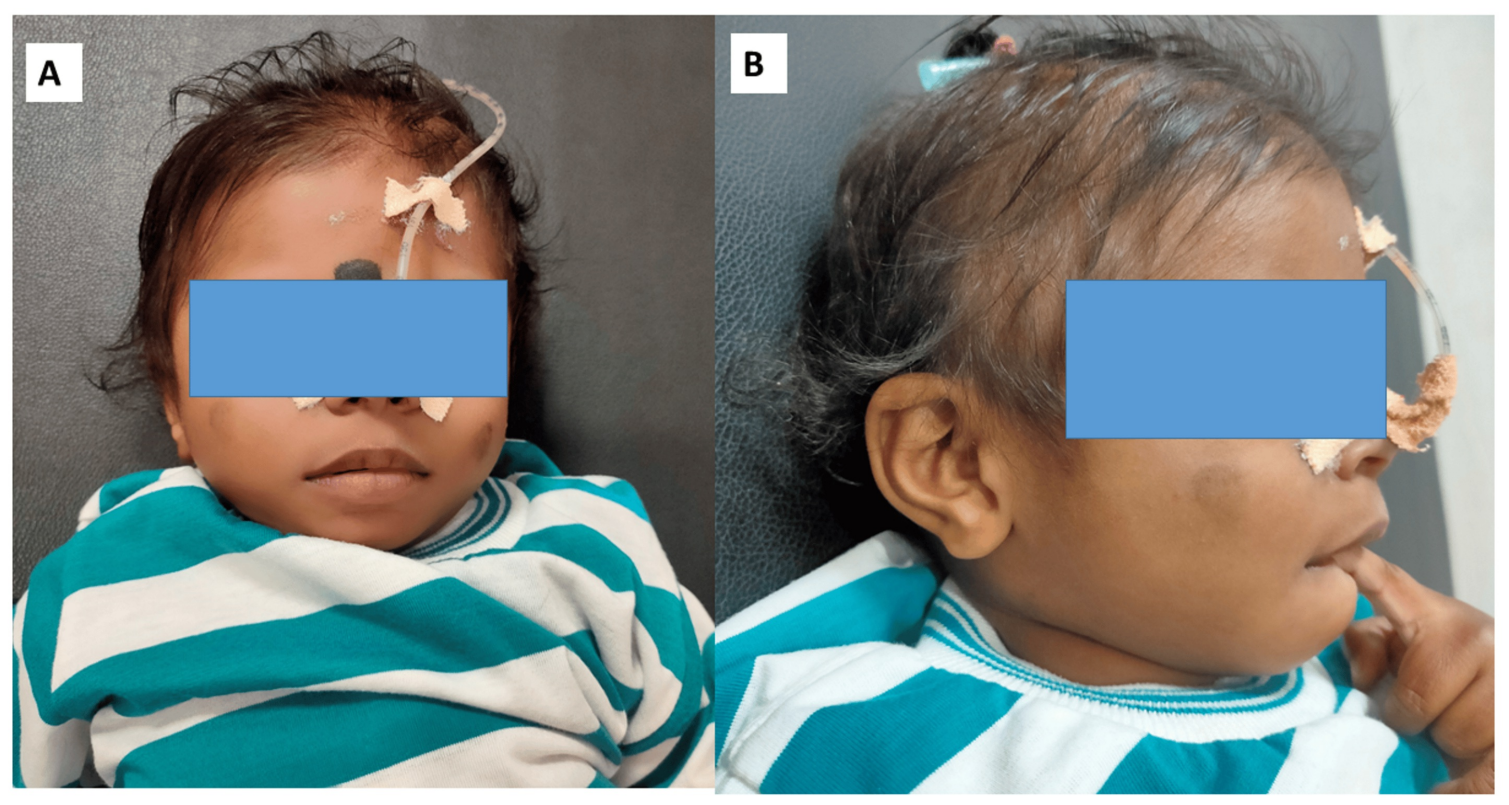
Facial photo of the affected patient, frontal (A) and lateral view (B) The frontal (A) and lateral (B) views of the face of the patient show typical Williams syndrome features: “elfin facies”, and a nasogastric tube is seen in situ. Written informed consent has been taken for publication of the facial photograph.

He achieved social smile and cooing at 2 months, head control at 5 months, then following the development of repeated myoclonic seizures (several seizures per day) regressed. At 10 months of age, he could not control his head, lost eye contact, and social smile, could coo occasionally, and responded to sounds but visual tracking was less. On examination, he was 69 cm long (- 1.9 SD). He displayed facial features suggestive of Williams syndrome (broad forehead, bitemporal narrowing, periorbital fullness, short nose, broad nasal tip, malar flattening, thick upper and lower lips, wide mouth, large ears) (Figures 2A-2B).

A detailed neurological examination was performed. At resting posture he showed little spontaneous movements, on arm traction it could be extended more than usual, and arm recoil was less. When performing the scarf sign, the elbow could be positioned between the midline and opposite axillary line (40 to 60 degrees). The hand position was predominantly closed. Lower extremity tone was diminished. On traction of the leg, reduced flexion at the knee was observed. Leg recoil was diminished. The popliteal angle was 110 to 150 degrees. The plantar reflex was flexor. Diminished deep tendon reflexes were observed. On checking for primitive reflexes, palmar grasp and plantar grasp were present, the Moro reflex was absent, the asymmetric tonic neck reflex was fairly integrated, and the positive support reflex was absent. On positioning in the supine position, the head was in the midline, visual tracking movements and midline activities were absent. On pull to sit, complete head lag was seen. He could initiate turning over to one side but could not roll over completely. On lying prone, head lifts were reduced but he could momentarily hold his head but trunk extension was absent. He required manual assistance to get the elbows under the shoulders. He maintained shoulder in abduction, elbow, and fingers in flexion, and forearm weight-bearing was absent. On being placed in supported sitting, his lower back was rounded and he could not support his head in a vertical position. His legs could not bear his weight on being placed in a standing position. He did not reach for any toys. Visual fixation and tracking were poor. There was a lack of eye-hand coordination.

The investigations performed were as follows. Electroencephalography was abnormal and showed intermittent spike-wave and polyspikes-wave discharges over bilateral posterior head regions during sleep. Estimation of amino acids and acylcarnitine analysis by tandem mass spectrometry showed low free carnitine (4.29 micromol/L, normal range 9-69). This was either suggestive of secondary carnitine deficiency due to chronic medication or loss in urine or due to primary carnitine deficiency/carnitine uptake disorder. Two-dimensional echocardiography showed moderate pulmonary valve stenosis. MRI of the brain showed mild thinning of the corpus callosum and no other abnormality.

Genomic DNA extracted from blood was used to perform targeted gene capture using a custom capture kit and the libraries were sequenced to mean >80-100X coverage on the Illumina sequencing platform. The sequences obtained were aligned to the human reference genome (GRCh38.p13) using Sentieon aligner and analyzed using Sentieon to remove duplicates, recalibration, and re-align indels. Sentieon haplotype caller was used to identify variants that are relevant to the clinical indication. Gene annotation of the variants was performed using the variant effect predictor (VEP) program against the Ensembl release 99 human gene model. In addition to SNVs and small indels, copy number variants (CNVs) were detected from targeted sequence data using the ExomeDepth (v1.1.10) method. This algorithm detects rare CNVs based on a comparison of the read-depths of the test data with the matched aggregate reference dataset. Clinically relevant mutations were annotated using published variants in literature and a set of disease databases - ClinVar, OMIM, GWAS, HGMD (v2020.2), and SwissVar. Common variants were filtered based on allele frequency in 1000Genome Phase 3, gnomAD (v2.1), EVS, dbSNP (v151), 1000 Japanese Genome, and our internal Indian population database. Non-synonymous variants effect was calculated using multiple insilico algorithms. Only non-synonymous and splice site variants found in the coding regions were used for clinical interpretation. A heterozygous contiguous deletion of size 1.431 megabases, was detected at the genomic location chr7:g.(?_73303398)_(74733981_?) (GRCh38 format). This was consistent with the clinical diagnosis of Williams syndrome. The genes included in the deleted region were: *GTF2IRD2, MIR590, CASTOR2, RCC1L, GTF2IRD2B, LIMK1, CLIP2, EIF4H, GTF2IP1, ELN, GTF2I, RFC2, LOC101926943, SPDYE12P, NCF1, LAT2, STAG3L2, GTF2IRD1, MIR10525, PMS2P5, NCF1C*. In addition, a novel homozygous missense variation in the exon 8 of the *TNK2 *gene (c.1066T>C; chr3:195605412 A>G, Depth:97, NM_001382273.1) (GRCh38 format) that results in the amino acid substitution of arginine for cysteine at codon 356(p.Cys356Arg). The variation has not been reported in the 1000 genomes, gnomAD databases. The insilico tools predicted the variant as mostly deleterious. The insilico predictions of pathogenicity as given by the Franklin site are given in Table 1 (https://franklin.genoox.com/clinical-db/variant/snp/chr3-195605412-A-G?app=assessment-tools).

**Table 1:** Insilico analysis for the 𝘛𝘕𝘒2 gene variant p.Cys356Arg using Franklin Online software The variant in *TNK2 *gene p.Cys356Arg was predicted to be deleterious by most *in-silico* algorithms.

Name of insilico software	Prediction of variant	Score
Franklin	Deleterious	0.86
Rare exome variant ensemble learner (REVEL)	Deleterious (moderate)	0.87
AlphaMissense	Deleterious (strong)	0.999
Varity	Deleterious	0.98
Mutation Assessor	Medium impact	3.1
Sorting intolerant from tolerant (SIFT)	Deleterious (supporting)	0
MutationTaster	Deleterious	1
Functional analysis through hidden Markov models (FATHMM)	Uncertain	-1.85
Deleterious annotation of genetic variants using neural networks (DANN)	Deleterious	1
Logistic regression-based ensemble prediction tool (MetaLR)	Deleterious	0.81
PrimateAI (artificial intelligence tool)	Deleterious (supporting)	0.84
BayesDel (deleteriousness meta-score)	Deleterious (moderate)	0.4
Splice AI tool	Benign	0
Genomic evolutionary rate profiling (GERP)	Uncertain	5.12
GenoCanyon	Deleterious	1
Fitness consequence tool (FitCons)	Deleterious	0.71

**Figure 3:**
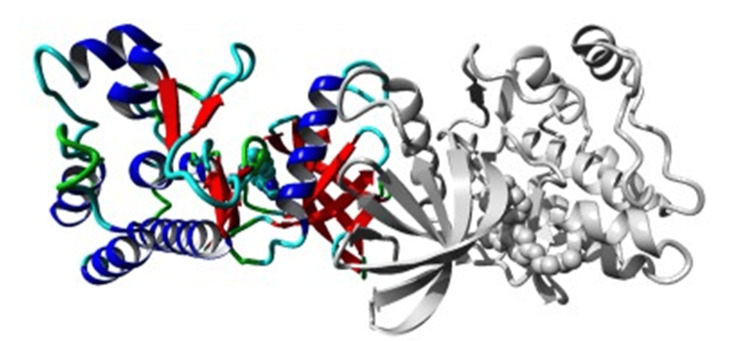
Overview of the protein 𝘛𝘕𝘒2 in ribbon presentation The protein is colored by element; α-helix=blue, β-strand = red, turn=green, 3/10 helix=yellow, and random coil=cyan. Other molecules in the complex are colored gray when present.

The variation lies in the protein tyrosine kinase of the TNK2 protein. The insilico analysis was also performed using HOPE (Have Your Protein Explained) software to determine the structural effects produced by the variant (www3.cmbi.umcn.nl/hope/input/). The HOPE analysis suggested the following: the mutant residue arginine is larger than cysteine which is buried in the core of the protein, hence it may not fit. Also, arginine is less hydrophobic than cysteine. Arginine is positively charged whereas cysteine is neutral amino acid. Thus, the variant may affect the molecular interactions in the protein. The variant is likely to abolish the function of the protein kinase domain. The residue is relatively well conserved in evolution. The image overview of TNK2 protein is shown in Figure 3.

**Figure 4:**
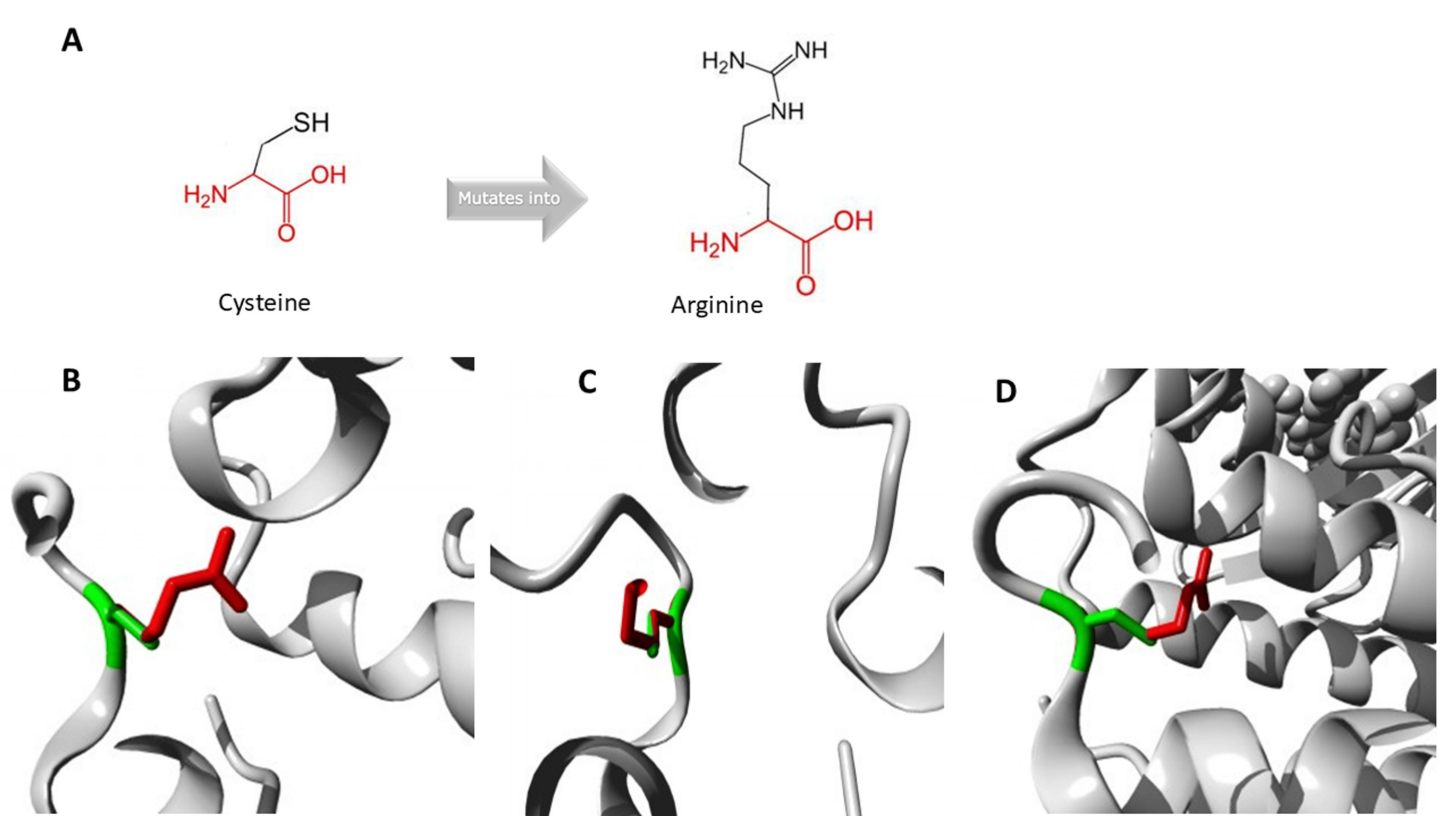
3D modeling of the variant in 𝘛𝘕𝘒2 gene by HOPE online software A shows the schematic structures of the original (left) amino acid cysteine and the mutant (right) amino acid arginine. The backbone, which is the same for each amino acid, is red. The side chain, unique for each amino acid, is black. B-D show a close-up of the mutation in the protein in ribbon presentation from different angles. The protein is gray. The side chains of both the wild-type and the mutant residue are green and red, respectively.

The schematic representation of the amino acid substitution is seen in Figure 4A and the images of the close-up of the mutation as shown by HOPE analysis are seen in Figures 4B-4D.

Video 1 shows a close-up of the mutation as shown by HOPE analysis from different angles. 

**Video 1:** 3D modeling of the 𝘛𝘕𝘒2 gene variant p.Cys356Arg using HOPE software A rotating close-up view of the *TNK2 *gene variant p.Cys356Arg. The protein is colored gray, and the side chains of both the wild-type and the mutant residue are shown and colored green and red, respectively.

Video 2 shows a close-up of the mutation as shown by HOPE analysis in alternating views.

**Video 2:** 3D modeling of the 𝘛𝘕𝘒2 gene variant p.Cys356Arg using HOPE software A close-up of the *TNK2 *gene variant p.Cys356Arg. The protein is colored gray, and the side chains of both the wild-type and the mutant residue are shown and colored green and red, respectively. The animation shows an alternating wild-type side chain and the mutant side chain.

The results were counseled to the family. Parental testing for the *TNK2 *gene variant was advised, but the parents could not afford the same. He was treated with oral valproate 30 mg/kg/day and clobazam 0.3 mg/kg/day with a decrease in the frequency of seizures. He was also prescribed oral carnitine (50 mg/kg/day). Neurodevelopment therapy was initiated to improve trunk control, head orientation in a vertical position, facilitation of active functional rotation in the transverse plane, facilitating active weight shifts through weight transferring activities, and sensory-motor play activities.

## Discussion

Epilepsy has been uncommonly described in association with Williams syndrome cases. Typically, 95 % of Williams syndrome cases have a microdeletion of 1.5 to 1.8 megabases at the 7q11.23 locus. In the rest 5 %, atypical larger deletions are present. The elastin *ELN *gene is the most important in the Williams syndrome microdeletion syndrome deleted region [1,2]. Patients with larger deletions including *HIP1, MAGI2*, or *YWHAG1* genes have more severe neurological phenotypes and seizures [7]. The *HIP1, MAGI2*, and *YWHAG1* genes were not included in the deleted region in our patient. However, a study by Nicita et al. neither found any significant association with the larger size of the deletion nor any specific gene at the locus [8]. They showed that both typical and atypical deletions are possible in Williams. They published nine cases of Williams syndrome with epilepsy. Three cases had the typical size of deletion, two cases had atypical sizes (1.92 MB and 2.28 MB, respectively), and in four cases, size was not reported (FISH study only). Our patient had a smaller (atypical) deletion of 1.45 MB. As per the study of Alesi et al., our patient had the crucial genes GTF2IRD1 and GTF2I deleted and hence, intellectual disability and facial phenotype of Williams syndrome were expected [9]. Deletion of the ELN (elastin) gene in our patient correlated with the finding of pulmonary valve stenosis. Nicita et al. [8] also reviewed 18 previously published cases of Williams syndrome and epilepsy. Eight out of 18 cases had infantile spasms with hyps arrhythmia (West syndrome), six cases had generalized tonic-clonic/tonic seizures, two cases had absence seizures, one case had ictal apneas and in one case seizure type was not known. Age at onset ranged from 2 months to 2 years. Neuroimaging was abnormal in 4 out of 17 cases. One case had mild cortical atrophy (Mizugishi et al.), another had craniosynostosis (Del Morimoto et al.) and yet another had periventricular nodular heterotopia, cerebellar hypoplasia, enlarged retro cerebellar space (Kogelenberg et al.) [10,11,12]. The case report by Okamoto et al. had congenital hydrocephalus, corpus callosal agenesis, thin cortex, and pontocerebellar hypoplasia [13]. Muhle (2017) reviewed 592 retrospective cases of Williams syndrome and found epilepsy in six cases (1 %), four cases had non-lesional West syndrome (infantile spasms) and two cases had lesional focal epilepsy due to hypoxic brain injury and trauma, respectively. Age at onset in West syndrome was between 3.5 and 7.5 months. Long-term epileptic therapy (ACTH, cortisone, nitrazepam, topiramate) was terminated between 8 and 24 months of age. None of these cases had exome sequencing done to investigate the possibility of other genetic factors (https://aesnet.org/abstractslisting/williams-syndrome-and-epilepsy--a-retrospective-analysis-of-592-patients). Popp et al. identified a child with Williams syndrome and refractory infantile spasms due to denovo truncating mutation c.1200del, p.Lys401Serfs*25 in GABRA1 gene (revealed by trio exome sequencing), thus indicating that genes other than that included in the deleted region could play a significant role in the development of epilepsy in Williams syndrome cases [14].

A review of cases published with *TNK2 *gene mutations was also performed. Hitomi et al. identified three siblings with infantile-onset epilepsy and cognitive regression [5]. The children developed focal seizures with automatisms, and secondary generalization at the age of onset around 19-35 months. Early development was normal, onset of epilepsy was associated with cognitive regression as in our case. MRI brain and interictal EEG were normal. Patients required multiple antiepileptics. A homozygous missense mutation in *TNK2 *gene p.Val716Met was found in all three sibs with parents being heterozygous for the variant. This variant interferes with the binding of TNK2, a brain-expressed tyrosine kinase with ubiquitin ligases NEDD1 and NEDD4 leading to loss of degradation after epidermal growth factor stimulation. Hitomi et al. sequenced the *TNK2 *gene in further 110 patients with infantile-onset epilepsy and identified six novel heterozygous variants in the *TNK2 *gene. These variants include p.Ala32Thr, p.Arg116Trp, p.Arg250Ter, p.delPhe590, p.Pro584HisfsX16, p.Pro752Ser which were also seen in heterozygous unaffected parents. Hitomi et al. thus, proposed that *TNK2 *gene mutation could be an autosomal recessive genetic syndrome of early onset epilepsy with cognitive regression.

Mao et al. reported two more unrelated cases of epilepsy and *TNK2 *gene mutation [6]. The first case was a girl with infantile spasms and hypsarrhythmia since 13 months of age with compound heterozygosity for *TNK2 *gene mutations c.2860 G>T or p.Glu954Ter and c.3004 G>T or p.Gy1002Cys. The second case was a girl with infantile spasms since 11 months of age and compound heterozygosity for mutations c.1705G>A or p.Thr569Ala and c.2243G>A or p.Arg748Gln. Our case is the fourth reported case of association of epilepsy with a biallelic mutation in the *TNK2 *gene. Insilico analysis performed using HOPE software and various tools showed a deleterious effect of the variant on the structure and function of the protein TNK2.

The patient’s family was thus counseled that the deletion causative of Williams syndrome was a likely sporadic event not inherited from the parents, whereas the homozygous *TNK2 *gene variant could be a possible cause of epilepsy and is likely inherited from the parents in an autosomal recessive manner. Thus, the recurrence risk of Williams syndrome in the next child of the couple was negligible but the recurrence risk of *TNK2 *gene-related epilepsy was likely to be 25 % in every pregnancy.

## Conclusions

The association of epilepsy and Williams syndrome is reported although the incidence of seizures in Williams syndrome is not greater than in the general population. Other genetic causes should be looked for in cases with early onset epilepsy/epilepsy requiring multiple antiepileptics. *TNK2 *gene mutation could be a rare cause of epilepsy, more cases are needed to confirm this association. Genetic testing such as exome sequencing is essential for genetic diagnosis and counseling. Genetic counseling is necessary to understand the nature of the genetic disease, inheritance pattern, treatment options, recurrence risk in future sibs, and the possibility of prenatal diagnosis and preimplantation genetic diagnosis to prevent recurrences in the next child.
